# Ciliated Foregut Cyst of the Pancreas

**DOI:** 10.1155/1998/10546

**Published:** 1998-12

**Authors:** Imtiaz A. Munshi, Eduardo Parra-Davila, Victor J. Casillas, Danny Sleeman, Joé U. Levi

**Affiliations:** 1 Department of Surgery University of Miami Medical Center Miami Florida USA; 2 Department of Radiology University of Miami Medical Center Miami Florida USA

## Abstract

Cystic lesions of the pancreas are relatively uncommon. We describe the case of a young man with a complex cystic mass located within the head of the pancreas. The patient underwent exploration with resection of the mass. Pathology revealed a ciliated epithelial cyst, a rare cystic lesion of the pancreas.


*HPB Surgery*, 1998, Vol. 11, pp. 117–119
Reprints available directly from the publisher
Photocopying permitted by license only

© 1998 OPA (Overseas Publishers Association) N.V.
Published by license under
the Harwood Academic Publishers imprint,
part of The Gordon and Breach Publishing Group.
Printed in Malaysia.

### *Case Report*

### Ciliated Foregut Cyst of the Pancreas

IMTIAZ A. MUNSHI[a], EDUARDO PARRA-DAVILA[a],
VICTOR J. CASILLAS[b], DANNY SLEEMAN[a,*] and JOE U. LEVI[a]

[a] *Department of Surgery;* [b] *Department of Radiology, University of Miami Medical Center Miami, Florida*

*(Received 1 June 1997)*

**Cystic lesions of the pancreas are relatively uncommon. We describe the case of a young man with a complex cystic mass located within the head of the pancreas. The patient underwent exploration with resection of the mass. Pathology revealed a ciliated epithelial cyst, a rare cystic lesion of the pancreas.**

*Keywords:* Ciliated pancreatic cyst, duplication cyst, bronchogenic cyst

### INTRODUCTION

Cystic lesions of the pancreas are relatively uncommon. They are classified by the presence or absence of an epithelial lining. True cysts of the pancreas contain an epithelial lining whereas, false cysts contain a fibrous lining. True cysts are further subdivided into congenital and acquired cysts (Tab. I). We describe the fifth case in the English language literature of a rare type of congenital cyst of the pancreas, a ciliated foregut cystic lesion. A brief review of the literature is provided.

### CASE REPORT

The patient, a 33 year old white man, had been in excellent health until recently when he experienced severe epigastric abdominal pain radiating to the back not associated with nausea, vomiting, weight loss, or jaundice. Physical examination was significant for slight epigastric fullness with minimal tenderness to deep palpation. Diagnostic work-up included abdominal ultrasound examination which demonstrated a multilocular, cystic mass involving the head of the pancreas without evidence of biliary or pancreatic ductal dilatation. Further diagnostic work-up included a computed tomographic scan confirming the presence of a multilocular, cystic mass in the neck and head of the pancreas, this mass was inseparable from the celiac artery and the inferior vena cava (Fig. 1). The patient underwent exploratory laparotomy. The mass was attached to the uncinate process and was adherent to the celiac artery and the inferior vena cava. It was dissected from the celiac artery

*Corresponding author.

TABLE I Classification of pancreatic cysts

| | |
|---|---|
| A. Congenital | |
| | single true cyst |
| | polycystic kidney disease |
| | cystic fibrosis |
| | von Hippel-Lindau disease |
| B. Inflammatory | |
| | pseudocyst |
| | retention cysts |
| | abscess |
| | echinococcus |
| C. Neoplastic | |
| | 3. Benign |
| | microcystic adenomas (serous) |
| | cystic teratomas |
| | 2. Malignant or potentially malignant |
| | mucinous cystic neoplasm |
| | solid and papillary epithelial neoplasm |
| | necrotic islet cell tumor |
| | necrotic anaplastic or adenocarcinoma |
| | lymphoma |

and the inferior vena cava and transected from the uncinate process, leaving most of the uncinate process intact. His operative and post-operative courses were uneventful, and he was discharged on post-operative day seven.

Examination of the specimen revealed an irregular brown-tan colored, soft tissue mass covered by fibroadipose tissue, measuring 5.4×4.3×2.1 centimeters (cm). Cut section of the specimen revealed multiloculated cysts, ranging from 0.3 to 1.2 cm in greatest dimension. Most cysts had a smooth, glistening epithelial lining and were filled with a grey mucinous fluid.

### DISCUSSION

Ciliated foregut cysts of the pancreas are an unusual type of cyst of the pancreas. Four cases have been reported in the English language literature [1–4]. In contrast to simple, unilocular congenital cysts of the pancreas these cysts tend to occur in adults. Ciliated foregut cysts of the pancreas are analagous to a bronchogenic cysts of the lung or to enterogenous (duplication) cysts of the proximal gastrointestinal tract. These cysts contain smooth muscle with a ciliated columnar or pseudostratified epithelial lining. The cysts contain material ranging from clear serous fluid to milky white or brown viscid, mucoid material with abundant lipid and

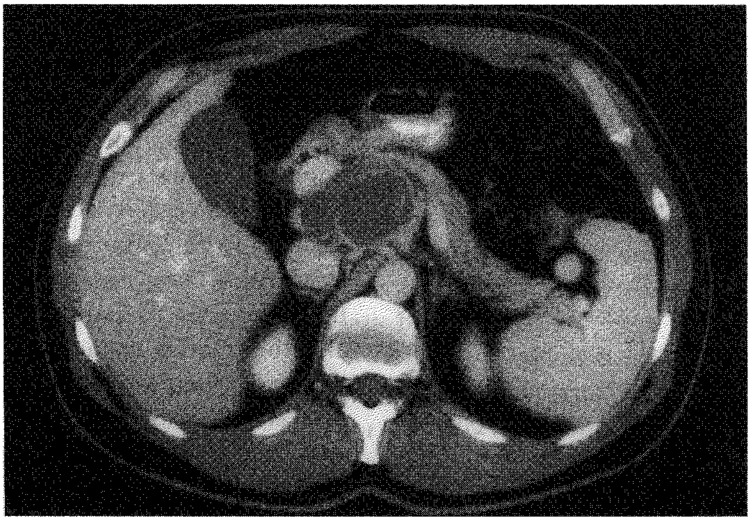

FIGURE 1 Computed tomography demonstrated a multilocular, well circumscribed cystic mass in the neck and head of the pancreas.

protein content [2, 3]. One report in the literature describes the presence of increased concentrations of carcinoembryonic and CA-125 antigens, features typical for a mucinous cystic neoplasm of the pancreas [3]. However, they are considered benign.

The differential diagnosis of cystic lesions of the pancreas includes several benign congenital cystic lesions, inflammatory and cystic neoplasms (Tab. I). Histologic analysis allows differentiation between many of the entities. Ciliated foregut cysts are differentiated from true teratomas (dermoid cysts) by the presence of ciliated respiratory type epithelium, with well developed bands of smooth muscle, and bronchial type mucinous glands and the lack of skin appendages, teeth, cartilage, glial elements, or sebaceous material [5]. Lymphoepithelial cysts of the pancreas are distinguished from ciliated epithelial cysts by the presence of a characteristic keratinized squamous epithelium surrounded by lymphoid stroma [6]. Ciliated foregut cysts of the pancreas are developmental abnormalities, believed to have no evidence of malignant potential therefore, lack of evidence of metaplasia differentiates them from cystic neoplasms of the pancreas.

### *References*

[1] Pilcher, C. S., Bradley, E. L. and Majmudar, B. (1982). Enterogenous cyst of the pancreas. *American Journal of Gastroenterology*, **77**(8), 576–577.
[2] Kohzaki, S., Fukuda, T., Fujimoto, T., Hirao, K., Matsunaga, N., Hayashi, K., Irie, J. and Kondo, N. (1994). Case Report: Ciliated foregut cyst of the pancreas mimicking teratomatous tumour. *The British Journal of Radiology*, **67**, 601–604.
[3] Pins, M. R., Compton, C. C., Southern, J. F., Rattner, D. W. and Lewandrowski, K. B. (1992). Ciliated enteric duplication cyst presenting as a pancreatic cystic neoplasm: report of a case with cyst fluid analysis. *Clinical Chemistry*, **38**, 1501–1503.
[4] Lyon, D. C. (1969). Recurrent pancreatitis caused by peptic ulceration in an intrapancreatic gastric reduplication cyst. *British Journal of Clinical Practice*, **23**, 425–6.
[5] Assawamatiyanont, S. and King, A. D. (1977). Dermoid cysts of the pancreas. *The American Surgeon*, **43**, 503–504.
[6] Hisaoka, M., Haratake, J., Horie, A., Yasunami, Y. and Kimura, T. (1991). Lymphoepithelial cyst of the pancreas in a 65 year-old man. *Human Pathology*, **22**(9), 924–6.

### COMMENTARY

Most cystic neoplasms of the pancreas arise from the exocrine component of the gland, but are much less common than solid tumours. As pseudocysts almost always are secondary features to acute or chonic pancreatitis or are caused by an outflow obstruction, e.g., a cancer of the pancreas or ampullary region, the lesions most likely to be confused with congential cysts are cystadenomas or cystadenocarcinomas. The latter has a markedly better prognosis than noncystic adenocarcinomas, which is the reason why cystic neoplasms always should be removed if not convincingly proved to be pseudocysts. Preoperatively it may be possible to differentiate the congential cystic lesions of the pancreas in the von Hippel-Lindau syndrome from the other neoplasms by the history of the patient (the effects of the hamartomatous lesions of the central nervous system dominate the clinical illness), but otherwise the congential cysts are almost indistinguishable from those of the small locular pancreatic cystadenoma. Moreover, intraoperative differentiation is often not possible and therefore the entire lesion has to be carefully evaluated in order to arrive at a final diagnosis.

Ciliated forgut cysts are rarities that most pancreatologists have never even heard of. In the few cases presented so far it seems as if the clinical symptoms and signs – as in the present case – are unspecific and of little help for the right diagnosis. Due to the improved saftey of modern pancreatic surgery in most patients radical resection of a cystic lesion like this is favoured, because only if the cystic tumor is resected completely, a pathological classification is possible.

Åke Andrén-Sandberg
Department of Surgery
Lund University Hospital
Lund, Sweden

